# P01-09 State-wide implementation of the ‘Sport-Health Houses' program

**DOI:** 10.1093/eurpub/ckac095.009

**Published:** 2022-08-29

**Authors:** Antoine Noël Racine, Marie Mullot, Gautier Christèle

**Affiliations:** 1 Pôle Ressources National Sport Santé Bien-Etre, French Ministry of Sports and the Olympic and Paralympic Games, Vichy, France; 2 Sport Policy Development Office, French Ministry of Sports and the Olympic and Paralympic Games, Paris, France

**Keywords:** physical activity, sedentary behaviour, health promotion, chronic disease, network

## Abstract

**Issue and problem:**

In France according the group of age, 37% to 81% of the population are insufficiently active (ANSES, 2017). In addition, more than 40% of adults have prolonged sedentary behaviours (? 7 hours of day) (ANSES, 2017). Moreover, 7,6% of premature deaths in France could be attributable to physical inactivity (GOPA, 2021). In many territories, opportunities to adopt a physical active lifestyle need to be improved (IGAS, 2018). To tackle these major issues, the French government have implemented the Sport-Health Houses (SHH) program through the national sport health strategy 2019-2024.

**Problem description:**

SHH have been launched in 2019 across the country. SHH are places were communities are welcomed and informed about multiples benefits of physical activity and sport. SHH also offer opportunities to evaluate people's fitness and to refer them to a ‘sport health' program through their own resources or through local stakeholders network. How the SSH program have been implemented across the country? Did SHH reach inactive people? How SHH impacted communities?

**Results:**

Each year since 2019, the ministry of sport and the ministry of solidary and health have launched a call for project to local stakeholders to their organization become a SHH by complying selection requirements. In January 2022, 436 SSH were created on metropolitan and overseas territories with attention to the most vulnerable. Since 2020, beyond information about the benefits and opportunities to practice locally a physical activity, almost 697 000 inactive people were supported following a sport-health program of SHH in primary prevention of which 45 000 people in secondary or tertiary prevention. The impact evaluation of SHH to the communities is in progress

**Lessons:**

A strong national policy can support the local level to develop Health-Enhancing the Physical Activity (HEPA) promotion. Moreover, it seems to be particularly relevant to develop SHH in territories with social inequalities to attract people generally far away of an active lifestyle.

**Main messages:**

An approach linking the national and the local level is promising to develop HEPA.

